# From Challenges to Solutions: Mapping the Landscape of Medical Professionalism and Ethics in Pakistan

**DOI:** 10.12669/pjms.40.4.9346

**Published:** 2024

**Authors:** Kamran Sattar, Shaukat Ali Jawaid

**Affiliations:** 1 Dr. Kamran Sattar, MBB, FAcadMEd, AoME (UK), MMedEd, UoD (UK), PhD Scholar MedEd, USM (Malaysia) Department of Medical Education, College of Medicine, King Saud University, Riyadh, Saudi Arabia; 2 Shaukat Ali Jawaid Chief Editor Pakistan Journal of Medical Sciences Karachi, Pakistan. E mail: pjms@pjms.org.pk

Addressing the healthcare professionals through this communication is a great privilege and a sense of shared responsibility, hoping to shed light on the critical issues of the current state of medical professionalism and ethics in Pakistan. As we stand at the crossroads of tradition and progress, Pakistan’s healthcare scene is undergoing transformational changes, bringing both problems and opportunities for medical professionals.

Medical professionalism is the cornerstone that supports patient trust and the integrity of healthcare systems.^1^ It includes ethical behavior, clinical excellence, and social responsibility. The dynamics of medical professionalism in Pakistan, a country with a diverse cultural heritage and a fast-developing healthcare system, need close consideration. One of the most difficult tasks for Pakistan’s medical community is maintaining a fine balance between tradition and innovation. As medical knowledge expands and technology becomes more integrated into healthcare delivery, it is critical that our healthcare workers keep up with these changes while maintaining the timeless principles of empathy, compassion, and ethical integrity.

While medical professionals confront distinct obstacles in different areas and circumstances, including Pakistan, here are five potential challenges, their implications and potential solutions. Medical professionals, worldwide, confront distinct obstacles in different healthcare domains and circumstances. Pakistan is also not immune, hence we enlist five probable challenges, their implications and potential solutions, in terms of medical professionalism.

## 1. ETHICAL DILEMMAS

### Challenge

A huge challenge in Pakistan in the context of medical professionalism lies within the resource allocation. There surely is scarcity of healthcare resources with massive patient loads. Such situations create dilemmas for future healthcare personnel in the form of patients’ needs and prioritizing decision making for resources distribution. Another common issue arises for the healthcare professionalism while they interact with the pharmaceutical industry. These issues require a great deal of balance between medical ethics and the common healthcare needs within a diverse population.

### Impact

The inability to resolve these conflicts could cause moral discomfort and make it more difficult to provide a robust educational environment for students to prepare them for patient-centered care in future.

### Solution

Include ethics education and cultural competency in the medical curriculum. Construct case-based learning situations that take into account the population’s diverse cultural backgrounds. Promote candid conversations to assist students in learning how to navigate cultural differences while maintaining moral standards. National Bioethics Committee formed by the Government of Pakistan in 2003 has prepared practical, feasible Guidelines on Healthcare Professionals’ interaction with Pharma Trade and Industry. It is approved by the Pakistan Medical & Dental Council and the Federal Government which should be implemented in letter and spirit.^2^

## 2. LIMITED RESOURCES AND ACCESS TO HEALTHCARE

### Challenge

Pakistan’s healthcare system confronts resource constraints^3^,^4^, including a lack of healthcare workers, equipment, and facilities. This can have an impact on medical students’ capacity to learn today to give appropriate treatment to patients tomorrow.

### Impact

Medical students may struggle with concerns about resource allocation and may find it difficult to provide high-quality care in resource-constrained environments.

### Solution

Put in place training courses that emphasize resource optimization and effective resource utilization. Motivate students to get trained (early in their career) to nurture their own practical ideas for such challenges. Working in teams during volunteer work campaigns is an example. Encourage students to learn more as how to be able to distribute government spending on healthcare infrastructure to solve these problems and enhance accessibility in general. These steps shall ensure judicious use of scarce financial resources.

## 3. COMMUNICATION BARRIERS AND CULTURAL SENSITIVITY

### Challenge

It should be understood that, at its core, health education is a communication process, and that effective communication is necessary for learning to occur.^5^ Providing high-quality treatment requires effective communication with patients, their families, and other medical professionals. Medical students may face difficulties due to differences in communication styles, educational backgrounds, and language variety. Within multi-cultural societies, like Pakistan^6^ it can be difficult to strike a balance between cultural and religious beliefs and universal medical ethics. When religious or cultural views clash with medical students’ ethical standards, they may confront complex decisions.

### Impact

A misunderstanding can cause misconceptions, lower patient satisfaction, and make it more difficult for healthcare teams to work together.

### Solution

Include instruction in communication skills, with a focus on culturally sensitive communication, in the medical curriculum. Offer language competence courses to improve students’ capacity to interact with patients who speak different languages. Promote interdisciplinary cooperation among healthcare teams to enhance communication in general.

## 4. PROFESSIONALISM IN HIERARCHICAL STRUCTURES

### Challenge

Institutional hierarchy may pose a challenge to medical education.^7^ The hierarchical nature of institutional environments may make it difficult for medical students to voice their ideas and participate in decision-making. Their capacity to speak candidly and function as an advocate for patients may be impacted by this.

### Impact

A lack of empowerment and voice may result in missed chances for improving patient care and hampered leadership development among medical students.

### Solution

Foster an inclusive and open communication culture inside medical organizations. Implement mentorship programmes that allow students to interact with seasoned healthcare professionals while breaking down hierarchies. Encourage feedback methods that allow students to express themselves and participate in decision-making processes.

## 5. BURNOUT AND MENTAL HEALTH

### Challenge

The difficult nature of medical education and practice, along with the heavy educational load, can lead to burnout and mental health problems in medical students.^8^

### Impact

Burnout can impair patient care, limit empathy, and have a detrimental influence on medical students’ overall well-being. It also jeopardizes the long-term viability of the healthcare workforce.

### Solution

To raise mental health awareness, incorporate wellness and resilience programmes within the medical curriculum. To assist students in dealing with stress, create support systems such as counselling services and peer support groups. Encourage a work-life balance and self-care culture, and fight for institutional policies that address the issues that contribute to burnout.

Addressing these challenges requires a multifaceted approach involving educational interventions, institutional support, and systemic changes within the healthcare system. Providing medical students with the tools to navigate these challenges is essential for fostering a culture of professionalism in the medical field in Pakistan.

Let us embrace this opportunity to emphasize the ideals that constitute medical professionalism in Pakistan as we navigate the crossroads of tradition and progress. We contribute not only to our patients’ well-being but also to the progress of our wonderful profession in the service of humanity.
